# Recurring bacterial strains, subclusters, and the importance of practising lessons learned

**DOI:** 10.1017/S0950268824001195

**Published:** 2024-12-16

**Authors:** Craig W. Hedberg, Melanie J. Firestone, Jeff B. Bender

**Affiliations:** Division of Environmental Health Sciences, University of Minnesota, Minneapolis, MN, USA

The routine use of whole genome sequencing (WGS) for surveillance is ushering in a new era in our understanding of the epidemiology of *Salmonella.* The granularity of WGS allows for precise, laboratory-based case definitions that facilitate the successful investigation of small clusters of cases, even when those clusters are part of serotypes or clonal groups that exhibit relatively little variability [1]. Importantly, this precision allows clusters to be identified and investigated despite temporal and geographic distances between the cases. In addition, because microorganisms reproduce and change in orderly and measurable ways, differences in WGS patterns between closely related isolates and strains can be organized in ways that allow for the inference of evolutionary relationships between larger population groups [2]. The US Centers for Disease Control and Prevention (CDC) have begun to exploit the attributes of WGS in their identification of reoccurring, emerging or persisting (REP) strains of enteric bacterial pathogens (https://www.cdc.gov/ncezid/dfwed/outbreak-response/rep-strains.html). Many of these strains appear to be distributed across complex animal and environmental reservoirs.

Brandenburg et al. demonstrated some of the challenges posed by REP strains in their investigation of two multistate outbreaks caused by a persisting strain of *Salmonella* Hadar (REPTDK01) [3]. The first outbreak was identified in the spring of 2020 and ultimately involved 848 ill people from 49 states. Because initial interviews indicated that a high proportion of ill people reported contact with backyard poultry, and because backyard poultry has been identified as a seasonally reoccurring source for multistate outbreaks of human salmonellosis [4], the investigation rapidly focused on identifying types of poultry exposure and poultry purchase locations. Animal exposure information was obtained from 56% of the cases [3]. Of these, 73% reported contact with backyard poultry. Chickens and ducks were the most common poultry types purchased, with multiple purchase locations and multiple hatcheries serving those purchase locations. However, no recent purchases of new birds were reported for 39% of cases with backyard poultry contact. Thus, there appear to have been multiple sources of transmission contributing to the occurrence of the outbreak.

Early in 2021, a second, *S.* Hadar cluster including 34 cases from 15 states was identified [3]. These strains were genetically related to the previous backyard poultry outbreak strains, but also to strains of *S.* Hadar that the US Department of Agriculture’s (USDA) Food Safety Inspection Service (FSIS) had isolated from ground turkey samples tested during 2020. Thirteen (41%) ill people in this outbreak were specifically asked about consuming poultry products as well as exposure to backyard poultry. Of these, 62% reported consumption of ground turkey and none reported exposure to backyard poultry [3]. The outbreak strain was isolated from turkey samples that originated from 14 slaughter or processing facilities. No connections between the various backyard poultry or ground turkey sources were identified.

The occurrence of two sequential outbreaks caused by genetically related strains of *S.* Hadar with apparently distinct exposure sources raises numerous questions about how to best approach these investigations in the future. Brandenburg et al. suggest the need for enhanced molecular characterization methods, such as the analysis of the pangenome of *Salmonella* isolates and enhanced data collection through outbreak investigation and research to determine if these apparently distinct transmission pathways are linked, and if so, what reservoirs exist to allow these strains to persist [3].

Gerner-Smidt et al. addressed the need for a One Health approach to using WGS for the investigation of outbreaks associated with the zoonotic and environmental reservoirs that characterize REP strains [2]. They recommended initially looking for tightly related clusters differing by up to 10 alleles spanning a short period of time to identify potential point source subclusters before expanding case definitions to explore relationships between the subclusters and the larger outbreak. The investigations of the *S.* Hadar outbreaks began with the identification of clusters with fewer than 10 allele differences between isolates, but the case definition rapidly expanded as backyard poultry sources were suspected [3]. At least two subclusters with fewer than five allele differences between isolates from ill people and associated food (ground turkey) or animal (duck) and environmental sources were identified during the two outbreak investigations. A phylogenetic tree of 950 outbreak-associated isolates appears to contain at least six distinct subclusters that appeared to match with zero or very few allele differences [3]. Four of these clusters were in branches of the tree identified with backyard poultry and two were entirely or mostly identified with ground turkey [3]. It is not clear whether Brandenburg et al.’s traceback efforts focused on identifying the specific poultry or food sources for these distinct subclusters as recommended by the Council to Improve Foodborne Outbreak Response (CIFOR) *Guidelines for Foodborne Disease Outbreak Response* [5]. Following the traditional logic of outbreak investigation, it is these smaller, better-defined clusters that are more likely to be linked to a specific source of production. Including too many epidemiologically unrelated isolates in tracebacks may dilute the epidemiological signal and could result in a failed traceback analysis [2].

In the early stages of an investigation, hypotheses about possible sources are generated based on (1) known sources of the pathogen causing illness, (2) person, place, and time characteristics of outbreak-associated cases, and (3) case exposure assessment [6]. During the backyard poultry investigation, case exposure assessment appears to have dominated hypothesis generation. The high proportion of early cases reporting contact with backyard poultry became the primary hypothesis tested. Even though *S.* Hadar outbreaks had been previously associated with ground turkey and FSIS isolated outbreak-related strains of *S.* Hadar from routinely collected ground turkey samples, ill persons were not systematically interviewed about ground turkey consumption. Interestingly, only one-third of ill persons who reported consuming turkey also had contact with backyard poultry. This, and the results of FSIS sampling of ground turkey products, suggests that a ground turkey outbreak may have been nested within the backyard poultry outbreak. It is not possible to determine whether the identification of an unrecognized ground turkey outbreak could have led to FSIS regulatory actions or mitigation efforts by the industry to prevent a subsequent outbreak.

During the subsequent ground turkey-associated outbreak, Brandenburg et al. adapted their methods to systematically interview ill persons both for consumption of ground turkey and backyard poultry contact [3]. This has now been incorporated as a standard practice in subsequent investigations of these persistent strains to facilitate focal investigation of narrowly defined subclusters found within the larger distribution of isolates in the strain complex [3]. This is consistent with standard methods recommended in the CIFOR Guidelines [5].

Although there is a strong bias towards publishing positive outbreak investigation findings in a way that emphasizes the strength of the investigation, the practice of reviewing outbreak investigations to identify missed opportunities to prevent or limit transmission should also become a standard practice. An example of why this is important can be drawn from a highly successful investigation of a *S. Enteritidis* outbreak associated with commercially manufactured ice cream [7]. In 1994, an outbreak of *S. Enteritidis* infections was recognized by a sudden increase in isolates being submitted to the Minnesota Department of Health’s Public Health Laboratory. Because *S. Enteritidis* is a common serotype and there were no methods available at the time to further characterize the isolates, a ‘wait and see’ approach was taken to monitor the outbreak’s progress. New cases accumulated daily. Exactly three weeks after the first notification from the Public Health Laboratory, a case–control study was launched to determine the source of the outbreak. Within 48 h, a strong epidemiologic association implicated a particular brand of commercially distributed ice cream. Within the following 24 h, the ice cream production facility was shut down. A recall was initiated 48 h later. At the time, this public health intervention was notable because it was initiated based on the strength of the epidemiologic evidence alone. The first ice cream samples tested positive for *S. Enteritidis* one week later. Quantitative analysis of contaminated ice cream samples demonstrated low levels of contamination (~6 organisms per serving) and a survey of the company’s customers estimated a 6.6% attack rate among persons who consumed the implicated products [7]. Ultimately, 150 confirmed cases were reported in Minnesota with an estimated 224 000 illnesses occurring nationwide. The source of the outbreak was traced to contamination of ice cream premix in tanker trailers that had previously carried liquid egg. The FDA modified food product transportation rules to prevent future outbreaks of a similar nature [8].

Once the outbreak investigation was launched, it progressed rapidly with many positive outcomes. However, the three weeks between the initial notice of the potential outbreak and the initiation of the case–control study allowed three weeks of potentially preventable exposures to occur. There were 74 confirmed cases in Minnesota associated with exposures during this three-week time period. Thus, the ‘wait and see’ approach may have allowed the outbreak to double in size beyond what it could have been had the outbreak intervention occurred soon after the initial outbreak was recognized. Based on exposures subsequently reported by these early cases, the results of an earlier case–control study would have most likely produced the same results as the case–control study that was conducted. The primary unknown is whether regulatory agencies and the company would have responded aggressively to strong epidemiologic data presented in the context of a much smaller outbreak.

This example highlights the importance of pursuing outbreak investigations with a sense of urgency. Every investigation requires some balancing of priorities. The illnesses that could be prevented by prompt action should weigh heavily on the balance. During the heat of the investigation, choices are inevitably made based on incomplete information. Some of these choices may advance the investigation and some may delay it. Our obligation is to objectively review our methods, identify the implications of the choices we made, and call out approaches that may reduce the likelihood of repeating these missed opportunities.

The routine use of WGS is leading to a growing recognition of the importance of REP strains with complex animal and environmental reservoirs. However, even in complex reservoir systems, there are likely to be nodes within the production and distribution chains at which organisms are concentrated or amplified in a way that will produce a discrete outbreak that can be identified against the background of sporadic infections that arise from the broader reservoir [9, 10]. Investigating these discrete outbreaks may require restrictive case-definitions to identify potential contributing factors and prevention measures specific to these settings [2]. Such investigations may also provide insight into the structure of the reservoir systems and important pathways for transmission that may be amenable to novel control measures. As our food system becomes increasingly complex and the tools we have to investigate and respond to outbreaks advance, ensuring that our methods match the goals of the investigation is key to success.

Furthermore, investigations of REP strains raise questions about how we define what an outbreak is. A point source subcluster of cases may be investigated and reported as an outbreak. Multiple subclusters may be linked to a common food distribution chain and that result may be reported as a single outbreak. Multiple distribution chains may be linked to a commodity or region, with the whole event reported as an outbreak. The use of a single term, ‘outbreak’ to refer to multiple, distinct epidemiologic settings may be a source of confusion for public health officials, food regulators, food industries, and the general public trying to stay informed about food safety risks. For example, in 1998 the Minnesota Department of Health began investigating two separate, but concurrent outbreaks of shigellosis in restaurant settings [11]. In both outbreaks, ill food handlers were identified and were suspected to be sources of contamination. However, pulsed field gel electrophoresis (PFGE) subtyping of isolates from outbreak-associated illnesses demonstrated that the outbreaks were linked to each other, but not to strains of *Shigella sonnei* circulating in the community. Ingredient-specific analyses of menu items in each restaurant identified chopped parsley as a common ingredient. These outbreaks were subsequently linked to several similar outbreaks in restaurants across North America. Each of the individual outbreaks occurred in restaurants with similar parsley handling characteristics [11]. Contaminated parsley from the common source was widely distributed, but the outbreak was only recognized in restaurants where time and temperature abuse of chopped parsley allowed amplification of the contamination. Thus, each of these restaurant-level events could be viewed as separate outbreaks or as subclusters in the larger parsley-associated outbreak.

How outbreaks such as this are reported to the National Foodborne Disease Outbreak Surveillance System (FDOSS) matters because outbreak surveillance data are used by federal food safety agencies ‘to inform food safety decision-making and provide pathogen-specific direction for reducing foodborne illness’ [12]. These attribution efforts could be enhanced by clarifying and standardizing outbreak terminology based on outbreak definitions that are specific to the various levels of the food chain. REP strains appear to represent bacterial populations distributed widely across animal and environmental reservoirs. When these distributions intersect with nodes of the food system in which concentration or amplification can occur, detectable subclusters or outbreaks may occur. Viewing these events through a One Health lens is useful to highlight the complexity of their occurrence. As our awareness of these events grows, we must continue to critically review our methods in order to improve our practice.
